# Synthetic lethal interaction of cetuximab with MEK1/2 inhibition in *NRAS*-mutant metastatic colorectal cancer

**DOI:** 10.18632/oncotarget.11985

**Published:** 2016-09-12

**Authors:** Bernardo Queralt, Elisabet Cuyàs, Joaquim Bosch-Barrera, Anna Massaguer, Rafael de Llorens, Begoña Martin-Castillo, Joan Brunet, Ramon Salazar, Javier A. Menendez

**Affiliations:** ^1^ Department of Medical Oncology, Catalan Institute of Oncology, Girona, Catalonia, Spain; ^2^ Girona Biomedical Research Institute, Girona, Catalonia, Spain; ^3^ Department of Medical Sciences, Medical School, University of Girona, Girona, Spain; ^4^ ProCURE (Program Against Cancer Therapeutic Resistance), Metabolism & Cancer Group, Catalan Institute of Oncology, Girona, Catalonia, Spain; ^5^ Biochemistry and Molecular Biology Unit, Department of Biology, University of Girona, Girona, Spain; ^6^ Unit of Clinical Research, Catalan Institute of Oncology, Girona, Catalonia, Spain; ^7^ Department of Medical Oncology, Catalan Institute of Oncology, Bellvitge Biomedical Research Institute, L'Hospitalet de Llobregat, Barcelona, Spain

**Keywords:** colon cancer, KRAS, NRAS, cetuximab, MEK1/2

## Abstract

*KRAS* mutations are an established predictor of lack of response to EGFR-targeted therapies in patients with metastatic colorectal cancer (mCRC). However, little is known about the role of the rarer *NRAS* mutations as a mechanism of primary resistance to the anti-EGFR monoclonal antibody cetuximab in wild-type *KRAS* mCRC. Using isogenic mCRC cells with a heterozygous knock-in of the *NRAS* activating mutation Q61K, we aimed to elucidate the mechanism(s) by which mutant *NRAS* blocks cetuximab from inhibiting mCRC growth. *NRAS*^*Q61K*/+^ cells were refractory to cetuximab-induced growth inhibition. Pathway-oriented proteome profiling revealed that cetuximab-unresponsive ERK1/2 phosphorylation was the sole biomarker distinguishing cetuximab-refractory *NRAS*^*Q61K*/+^ from cetuximab-sensitive *NRAS*^+/+^ cells. We therefore employed four representative MEK1/2 inhibitors (binimetinib, trametinib, selumetinib, and pimasertib) to evaluate the therapeutic value of MEK/ERK signaling in cetuximab-refractory *NRAS* mutation-induced mCRC. Co-treatment with an ineffective dose of cetuximab augmented, up to more than 1,300-fold, the cytotoxic effects of pimasertib against *NRAS*^*Q61K*/+^ cells. Simultaneous combination of MEK1/2 inhibitors with cetuximab resulted in extremely high and dose-dependent synthetic lethal effects, which were executed, at least in part, by exacerbated apoptotic cell death. Dynamic monitoring of real-time cell growth rates confirmed that cetuximab synergistically sensitized *NRAS*^*Q61K*/+^ cellsto MEK1/2 inhibition. Our discovery of a synthetic lethal interaction of cetuximab in combination with MEK1/2 inhibition for the *NRAS* mutant subgroup of mCRC underscores the importance of therapeutic intervention both in the MEK-ERK and EGFR pathways to achieve maximal therapeutic efficacy against *NRAS*-mutant mCRC tumors.

## INTRODUCTION

Mutations in *KRAS* (35-45%) are a well-established predictor for lack of response to EGFR-targeted therapies in patients with metastatic colorectal cancer (mCRC), and are examined routinely to identify those patients unlikely to benefit from these therapies [1–4]. Recent studies have demonstrated that the evaluation of an extended panel of *RAS* mutations, including mutations in *NRAS*, can better define the patient population unlikely to benefit from anti-EGFR therapy while concomitantly improving the outcomes in a more highly selected *RAS* wild-type group [4–8]. However, although much is known about the prognostic and predictive roles of the highly prevalent *KRAS* mutations in mCRC, less is known about the role of the rarer *NRAS* mutations (3%) as a mechanism of primary resistance to EGFR-targeted therapies in *KRAS* wild-type mCRC.

Since they typically do not coexist in the same tumor [9–11], it is possible that mutations in *KRAS* and *NRAS* genes are functionally redundant as they could provide similar or identical oncogenic signals. However, recent molecular evidence supports the idea that mutations in *KRAS* and *NRAS* are not mutually exclusive; rather, they constitute molecular events that are specifically selected in response to significantly different tumorigenic contexts [12, 13]. In mice genetically engineered to express mutationally activated forms of *KRAS* and *NRAS* in the intestinal epithelium, mutant *KRAS* induces hyperproliferation of the colonic epithelium, which manifests as the appearance of a chronic intestinal hyperplasia [12]. Mutant *KRAS* therefore seems to enhance the transition from a benign adenoma to a malignant adenocarcinoma in a context of inactivation of the tumor suppressor gene adenomatous polyposis coli (*APC*). By contrast, mutant *NRAS* does not affect the initial homeostasis or tumor progression but inhibits the ability of intestinal epithelial cells to undergo programmed cell death in response to chronic exposure to apoptotic stimuli [13]. In this regard, it should be noted that both acute and chronic inflammation significantly contributes to colorectal cancer progression [14]. Accordingly, recent studies in genetically modified animals confirm that mutant *NRAS* might accelerate colorectal cancer development in the setting of inflammation [13]. At present, however, how and why the anti-apoptotic phenotype associated with activating mutations in *NRAS* can contribute to the origin, progression and response to targeted treatment of mCRC with anti-EGFR monoclonal antibodies such as cetuximab and panitumumab remains unknown.

*NRAS* is the least studied member of the RAS family of GTPases, and consequently the oncogenic properties associated with this isoform are not well characterized. Moreover, directly targeting oncogenic *NRAS* is extremely challenging for rational drug design, and no clinically available mechanism-based therapy for tumors with oncogenic *NRAS* mutations exists. We here envisioned that a careful characterization of the oncophenotype caused by the interaction of clinically relevant activating *NRAS* mutations with the phospho-proteome generated in response to EGFR-targeted therapies might facilitate the discovery of more effective therapies for the subgroup of patients with *NRAS*-mutated mCRC. We applied this approach to survey the changes in the phospho-proteome in an isogenic mCRC model (SW48 cells), in which one allele of the endogenous *NRAS* gene was edited to harbor an activating mutant c.181 C > A (*Q61K*). Using SW48 *NRAS*^*Q61K*/+^ cells, we herein describe a therapeutically targetable mechanism whereby mutant *NRAS* prevents cetuximab from inhibiting mCRC growth but is responsive to the development of an effective drug mix involving cetuximab and currently available MEK1/2 inhibitors.

## RESULTS

### Heterozygous knock-in of the *NRAS* activating mutation *Q61K* is sufficient to promote loss of sensitivity to cetuximab in a model of mCRC

**Figure 1:**
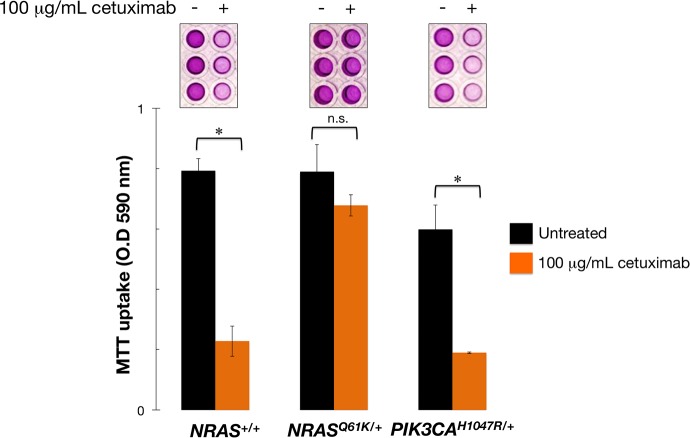
Mono-allelic activation of *NRAS* is sufficient to confer refractoriness to cetuximab in mCRC cells Cell viability of *NRAS*^+/+^, *NRAS*^*Q61K*/+^, and *PIK3CA*^*H1047R*/+^ SW48 cells cultured with 100 μg/mL cetuximab was assessed using an MTT assay. All assays were performed at least three times in triplicate. n. s. Non-significant differences were identified by Student's *t* test for paired values; ^*^P < 0.01 compared to control cells by Student's *t* test for paired values.

We utilized an SW48-based mCRC model to evaluate the impact of an activating *NRAS* mutation on the efficacy of the anti-EGFR monoclonal antibody cetuximab. To do this, we used SW48 colon cancer cell lines in which one allele of the endogenous *NRAS* gene contained a heterozygous knock-in of the c.181C > A activating mutation, resulting in an amino acid substitution from glutamine (Q) to lysine (K) at position 61. As expected, *NRAS*^*Q61K*/+^ cells were fully refractory to cetuximab-induced reduction in cell viability, whereas a strong reduction of cell viability was noted for parental *NRAS*^+/+^ cells in the presence of 100 μg/mL cetuximab (Figure 1). The isogenic introduction of the *PIK3CA* c.3140A > G (*H1047R*) activating mutation into *NRAS*^+/+^ cells, which results in an amino acid substitution at position 1047 in PIK3CA, from a histidine (H) to an arginine (R), failed to alter the sensitivity to cetuximab in SW48 mCRC cells (Figure 1).

### A low-scale proteomic analysis of *NRAS*-mutant mCRC cells reveals phosphorylation of MEK1/2 as the most relevant biomarker of resistance to cetuximab

To examine whether the differential effects of cetuximab on EGFR-dependent cell survival were related to constitutive changes in signaling components downstream of EGFR, we comprehensively surveyed the phosphorylation status of multiple intracellular kinases in *NRAS*^+/+^ and *NRAS*^*Q61K*/+^ cells in the absence or presence of cetuximab using low-scale semi-quantitative phospho-proteomics.

**Figure 2:**
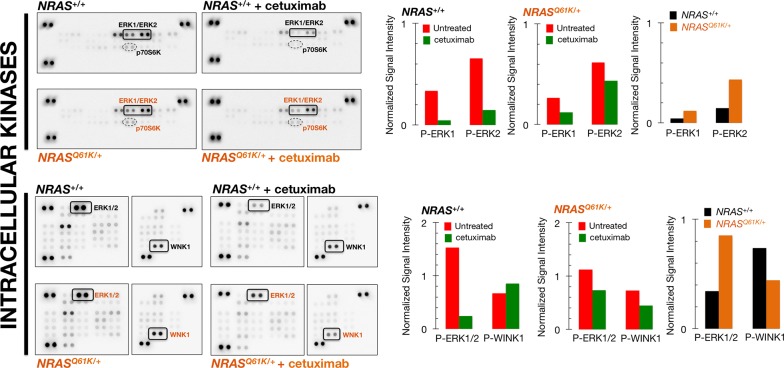
ERK1/2 activation is unresponsive to cetuximab resistance in *NRAS* mutant mCRC cells *Left panels.* Phospho-proteome profiling of mCRC cells in response to cetuximab. Total cell lysates (750 μg) from *NRAS*^+/+^ and *NRAS*^*Q61K*/+^ cells before and after treatment with 100 μg/mL cetuximab (48 h) were incubated on membranes of the phospho-proteomics platforms, human Phospho-MAPK (*top panels*; 23 different MAPKs and other serine/threonine kinases) and human Phospho-Kinase Arrays (*bottom panels*; 43 different kinases and 2 related total proteins), as described in “Methods and materials”. Figure shows representative phospho-proteome analyses. Equivalent results were obtained in two independent experiments. *Right panels.* Bar graphs show the results of densitometry analysis of the scanned phospho-arrays. Signal values include the background correction and the intensities normalization to the corresponding positive control values on each array.

Using the commercially available Proteome Profiler Human Phospho-MAPK Array kit (24 MAPK-related kinases), we found that *NRAS*^+/+^ cells exhibited a strong constitutive activation of ERK2 (T185/y187) and ERK1 (T202/Y204), which was markedly reduced in response to cetuximab treatment (Figure 2, *top panels*). By contrast, the constitutive activation of ERK2 and ERK1 remained largely unchanged in cetuximab-treated *NRAS*^*Q61K*/+^ cells (Figure 2, *top panels*). Thus, in the presence of cetuximab, cetuximab-refractory *NRAS*^*Q61K*/+^ cells exhibited an approximately 3-fold up-regulation in ERK1/ERK2 activity when compared to cetuximab-sensitive *NRAS*^+/+^ parental cells (Figure 2, *top panels*). Although less pronounced, the activation status of p70 S6 kinase (T421/S424) followed a pattern similar to that for ERK1/ERK2, i.e., whereas cetuximab treatment markedly reduced p70S6K activity in cetuximab-sensitive *NRAS*^+/+^ cells, phospho-active p70S6K was less responsive to cetuximab treatment in *NRAS*^*Q61K*/+^ cells (Figure 2, *top panels*).

Using the Human Phospho-Kinase Array, which is capable of simultaneously detecting the relative phosphorylation levels of 43 kinases and 2 related proteins, we confirmed that cetuximab-unresponsive ERK1/2 phosphorylation was the sole biomarker that distinguished cetuximab-refractory *NRAS*^*Q61K*/+^ from cetuximab-sensitive *NRAS*^+/+^ parental cells (Figure 2, *bottom panels*). Additionally, cetuximab treatment reduced the activation status of with no lysine kinase (WNK), a negative regulator of the activation of MEK/ERK [15, 16], in cetuximab-refractory *NRAS*^*Q61K*/+^ cells but not in cetuximab-responsive *NRAS*^+/+^ cells (Figure 2, *bottom panels*), further suggesting the central role of unresponsive ERK1/2 phosphorylation in the cetuximab-refractory phenotype of *NRAS* mutant cells.

### *NRAS*-mutant cells are more resistant to MEK1/2 inhibition

The phospho-proteomic signature of cetuximab-sensitive *NRAS*^+/+^ cells strongly suggested that the anti-proliferative mechanism of action of cetuximab in the SW48 model involved the deactivation of the MEK/ERK transduction cascade downstream of EGFR. The refractoriness of *NRAS*^*Q61K*/+^ cells to cetuximab, in turn, was consistent with the lack of inhibition of constitutively active MEK/ERK signaling in these cells. Given this, we examined the effects of four representative MEK1/2 inhibitors [17–20], namely binimetinib (MEK162, ARRY-162, a MEK inhibitor developed by Array Biopharma), trametinib (GSK1120212, Mekinist, a MEK inhibitor developed by GlaxoSmithKline), selumetinib (AZD6244, a MEK inhibitor invented by Array and developed by AstraZeneca), and pimasertib (AS-703026, MSC1936369B, a MEK inhibitor developed by Merck KgaC) on cell viability of *NRAS*^*Q61K*/+^ and *NRAS*^+/+^ cells.

**Figure 3:**
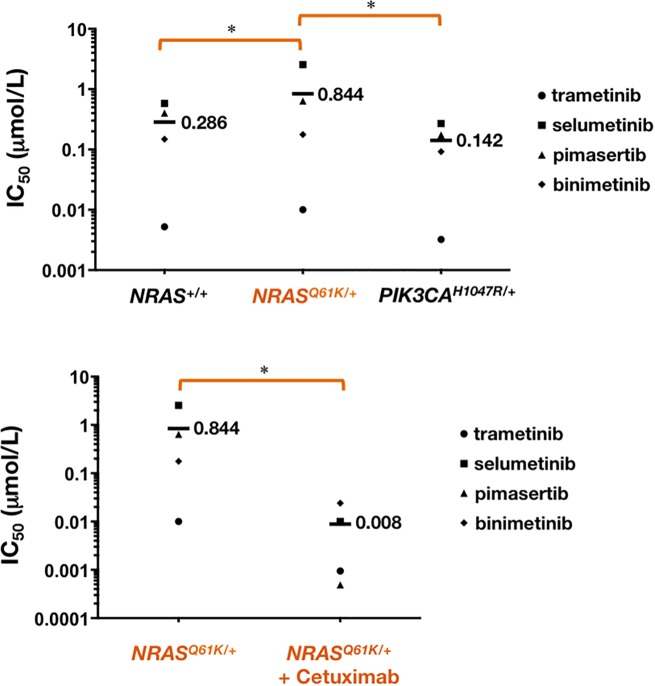
*NRAS* mutant mCRC cells are more resistant to MEK1/2 inhibitors Cell viability of *NRAS*^+/+^, *NRAS*^*Q61K*/+^, and *PIK3CA*^*H1047R*/+^ cells cultured with the MEK1/2 inhibitors binimetinib, trametinib, selumetinib, and pimasertib was assessed using an MTT assay. Concentrations causing 50% reduction in cell viability (IC_50_ values) were calculated in the absence (*top*) or presence (*bottom*) of cetuximab. ^*^P < 0.01 compared with control cells by ANOVA followed by Scheffé's multiple contrasts.

Scatter plots showing the interpolated IC_50_ values from dose-response curves revealed that *NRAS*^*Q61K*/+^ cells were less responsive to MEK1/2 inhibitors than *NRAS*^+/+^ cells (Figure 3). Thus, although the decrease in cell viability was dose-dependent and complete within the range of concentrations of MEK1/2 inhibitors employed, and the IC_50_ values were < 1 μmol/L for most of the MEK1/2 inhibitors, we observed that the IC_50_ values significantly increased by 2- and 5-fold when using trametinib and selumetinib, respectively, in *NRAS*^*Q61K*/+^ cells. The isogenic introduction of H1047R activating mutation at one of the endogenous PI3Kα loci, which is known to result in increased oncogenic PI3K pathway signaling, not only failed to alter the sensitivity to cetuximab (Figure 1) but also failed to promote resistance to MEK1/2 inhibitors in *NRAS*^+/+^ (Figure 3). Indeed, SW48 *PI3K*α^*H1047R*/+^ cells exhibited a significantly increased sensitivity (> 2-fold) to pimasertib and selumetinib when compared with SW48 *PI3K*α^+/+^ parental cells.

### Mutant *NRAS* activates a synthetic lethal interaction of MEK1/2 inhibitors combined with cetuximab

**Figure 4:**
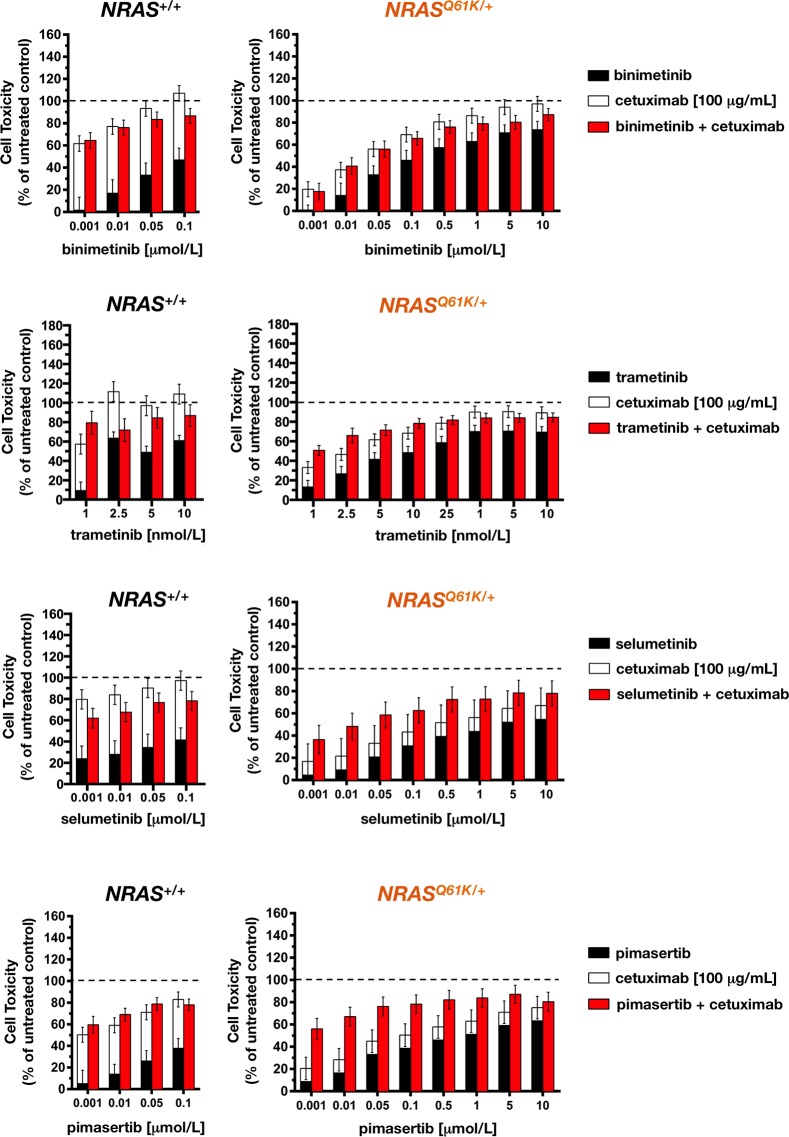
Cetuximab synergistically augments the toxicity of MEK1/2 against *NRAS* mutant mCRC cells Cells seeded in 96-well plates (2,000-3,000 cells per well) were cultured in triplicate with or without graded concentrations of MEK1/2 *plus/minus* 100 μg/mL cetuximab, which were not renewed during the entire period of cell exposure. For each pair of columns, the height of the left columns represents the sum of the toxic effect of each agent and, therefore, the expected toxicity if their effects were additive when used in combination. The total height of the right columns represents the observed toxicity when the agents were used in combination. The difference between the heights of the paired columns reflects the magnitude of antagonism or synergism on cell toxicity between MEK1/2 inhibitors and cetuximab in *NRAS*^+/+^ (*left panels*) and *NRAS*^*Q61K*/+^ (*right panels*) cells. Results are shown as mean (*columns*) ± SD (*error bars*) from at least three experiments in which triplicate wells were analyzed.

We next investigated the effects of combination treatment with MEK1/2 inhibitors and cetuximab. Whereas MEK1/2 inhibitors and cetuximab were highly effective as single agents in reducing cell viability in cetuximab-sensitive SW48 *NRAS*^+/+^ parental cells, as measured by the MTT reduction assay, concurrent exposure failed to show any synergistic effect. Thus, additive and less-than-additive (antagonistic) interactions occurred after combining graded concentrations of binimetinib, trametinib, selumetinib, and pimasertib with an optimal concentration of cetuximab (Figure 4, *right panels*), confirming that the combined treatment of MEK1/2 inhibitors with cetuximab could negatively interfere with cell growth inhibition in cetuximab-sensitive mCRC cells [21].

**Figure 5:**
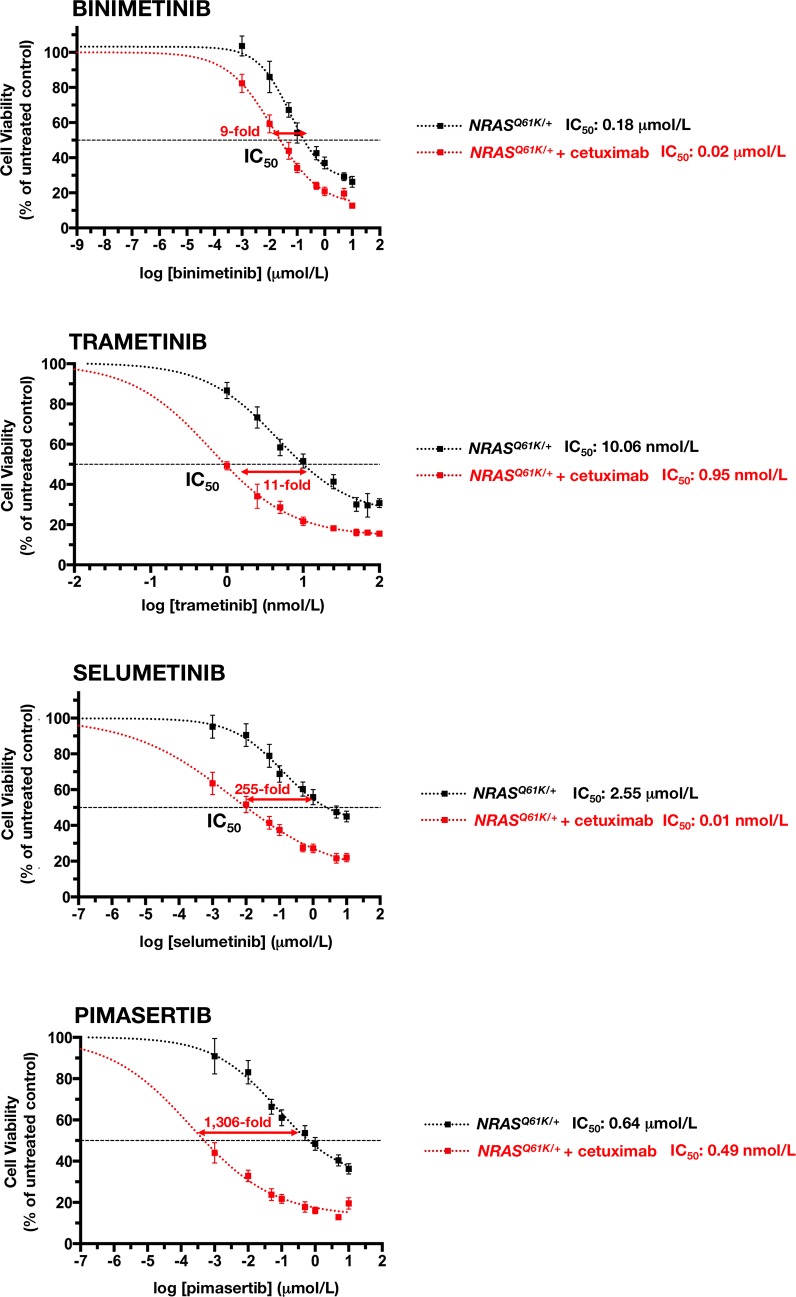
Cetuximab markedly sensitizes *NRAS* mutant mCRC cells to MEK1/2 inhibition The cell viability effects from exposure of *NRAS* mutant cells to MEK1/2 inhibitors were analyzed by generating concentration-effect curves as a plot of the fraction of unaffected (surviving) cells *versus* drug concentration. Dose-response curves were plotted as percentages of the control cells' absorbance (= 100%), which was obtained from wells treated with appropriate concentrations of agent vehicles that were processed simultaneously. IC_50_ values were designated for the concentrations of the agents decreasing absorbance values at 570 nm by 50%, as determined by interpolation using MTT-based colorimetric cell viability assays. Values are means ± SD from at least three experiments in which triplicate wells were analyzed. Sensitization factors were obtained by dividing IC_50_ values of MEK1/2 inhibitors alone by those obtained when cetuximab (100 μg/mL) was simultaneously supplemented.

In cetuximab-refractory *NRAS*^*Q61K*/+^ cells, however, the combined addition of any of the tested MEK1/2 inhibitors together with cetuximab resulted in an increase in cell toxicity, which was significantly higher than the additive value of the 2 drugs alone. Interestingly, the synergistic interaction was particularly evident at lower inhibitory concentrations of MEK1/2 inhibitors, whereas little impact was observed at the higher levels of cell suppression (Figure 4, *left panels*). Accordingly, a very different picture emerged when MEK1/2 single-agent dose-response curves in cetuximab-untreated *NRAS*^*Q61K*/+^ cells were compared with those when MEK1/2 inhibitors were combined with a largely ineffective concentration of cetuximab (eliciting < 20% inhibition of cell viability) (Figure 5). Co-exposure to cetuximab induced a conspicuous leftward shift in the dose-response curves of MEK1/2 inhibitors and, consequently, a manifest decrease in their IC_50_ values. Accordingly, 100 μg/mL cetuximab caused a 7-fold increase in *NRAS*^*Q61K*/+^ binimetinib efficacy, an 11-fold increase in *NRAS*^*Q61K*/+^ trametinib efficacy, a substantial 255-fold increase in *NRAS*^*Q61K*/+^ selumetinib efficacy, and a remarkable > 1,300-fold increase in *NRAS*^*Q61K*/+^ pimasertib efficacy.

### Cetuximab synergistically triggers apoptotic cell death with MEK1/2 inhibitors in *NRAS* mutant mCRC cells

**Figure 6:**
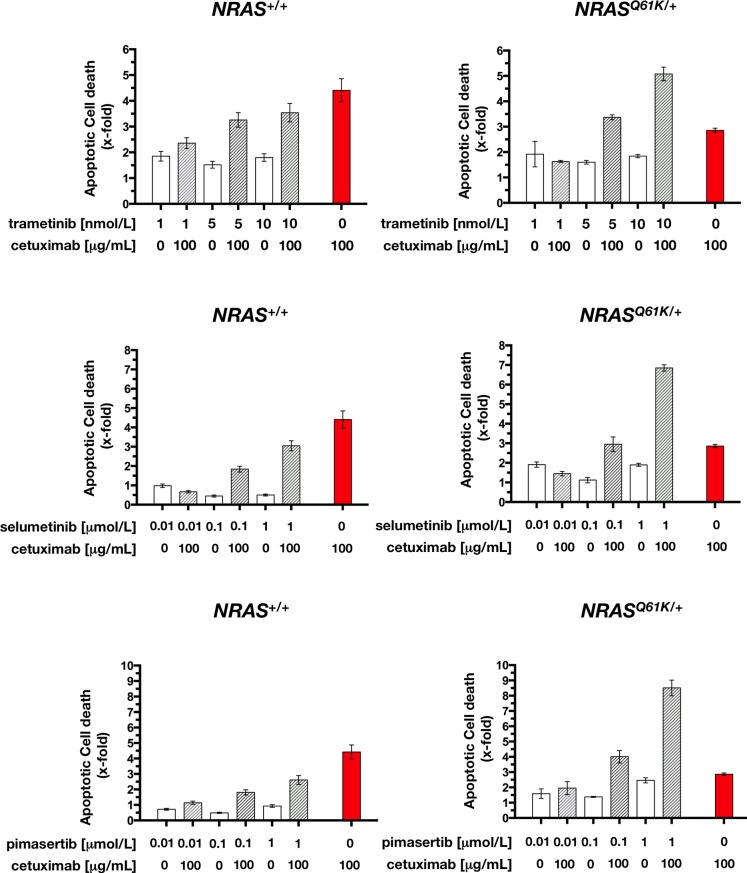
Cetuximab augments MEK1/2 inhibitor-induced apoptotic cell death in *NRAS* mutant mCRC cells Quantification of apoptosis-related cell death in *NRAS*^+/+^ (*left panels*) and *NRAS*^*Q61K*/+^ (*right panels*) cells in response to 72 h treatment with graded concentrations of MEK1/2 inhibitors in the absence or presence of cetuximab (100 μg/mL), was determined as described in “Materials and methods”. Results are shown as mean (*columns*) ± SD (*error bars*) from at least three experiments in which duplicate wells were analyzed.

To mechanistically explore the apparent synthetic lethal interaction between cetuximab and MEK1/2 inhibition in *NRAS*-mutant mCRC cells, we investigated the possibility that the decrease in cell viability was the result of an increase in apoptosis. Thus, *NRAS*^+/+^ and *NRAS*^*Q61K*/+^ cells were exposed to graded concentrations of the more efficient MEK1/2 inhibitors trametinib, selumetinib, and pimasertib for 72 h with or without 100 μg/mL cetuximab, and mono- and oligo-nucleosomes released into the cytoplasm by apoptotic cells was measured by ELISA. The addition of MEK1/2 inhibitors as single agents had almost negligible effects on apoptosis of *NRAS*^+/+^ and *NRAS*^*Q61K*/+^ cells. However, combination treatment of cetuximab with trametinib, selumetinib, or pimasertib resulted in enhanced apoptosis, which was significantly higher than the additive value of the 2 drugs alone (Figure 6). Interestingly, this synergism occurred for *NRAS*^*Q61K*/+^ cells and not for *NRAS*^+/+^ cells. Accordingly, combination of pimasertib and cetuximab resulted in ~3-fold-increase in apoptotic cell death than with pimasertib alone, and a 3.5-fold increase than with cetuximab alone. These findings suggest that combination of cetuximab with MEK1/2 inhibitors results in extremely high, dose-dependent synthetic lethal effects executed, at least in part, by exacerbated apoptosis.

**Figure 7:**
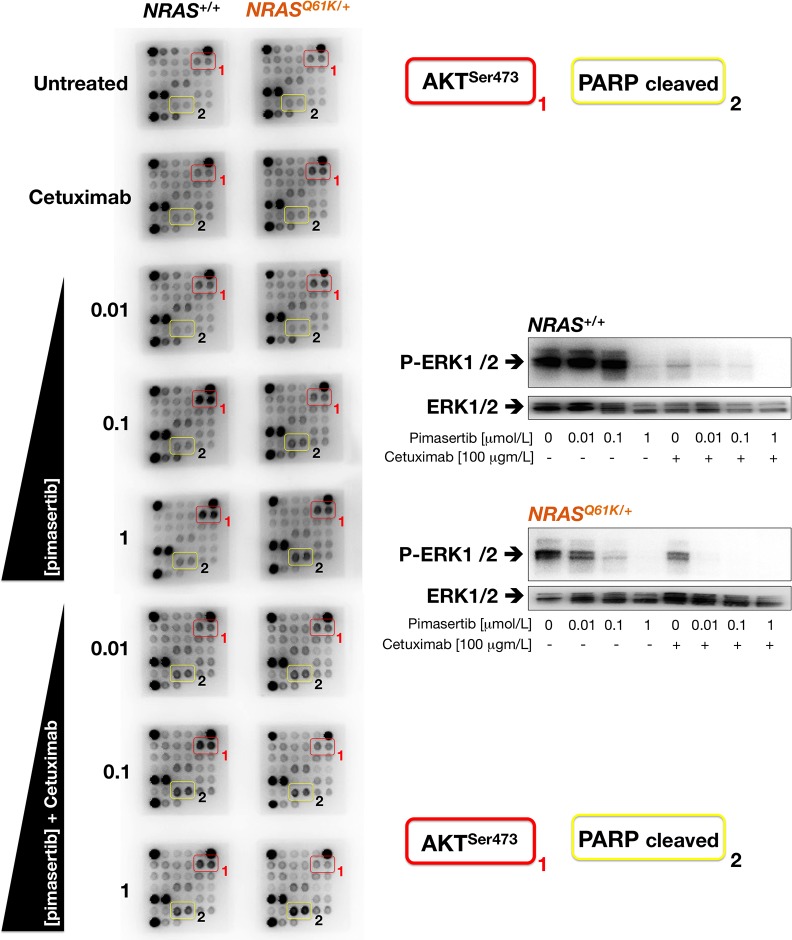
Cetuximab prevents AKT activation and promotes PARP cleavage in pimasertib-treated *NRAS* mutant mCRC cells *Left.* Figure shows representative chemiluminiscent array images from the PathScan Intracellular Signaling array kit showing key phosphorylated signaling nodes in *NRAS*^+/+^ (*left panels*) and *NRAS*^*Q61K*/+^ (*right panels*) treated with graded concentrations of pimasertib in the absence or presence of cetuximab. *Right.* Representative immunoblot analysis showing expression of phospho-ERK1/2 and corresponding total-ERK1/2 after exposure to graded concentration of pimasertib in the absence or presence of cetuximab.

To investigate underlying pathways, we used a commercially available slide-based antibody array to simultaneously assess 18 intracellular signaling molecules in their phosphorylated or cleaved state. When *NRAS*^*Q61K*/+^ and *NRAS*^+/+^ cells were treated with graded concentrations of pimasertib, AKT phosphorylation at Ser^473^ was higher in *NRAS*^+/+^ cells than in *NRAS*^*Q61K*/+^ cells (Figure 7). Co-exposure to cetuximab failed to completely abrogate the strong activation of AKT induced by pimasertib in *NRAS*^+/+^ parental cells. By contrast, addition of cetuximab was sufficient to prevent the weaker activation of AKT in pimasertib-treated *NRAS*^*Q61K*/+^ cells, which was accompanied by the evident activation of intracellular mediators of apoptosis, such as cleaved PARP protein (Figure 7). The inverse correlation of AKT activation and PARP cleavage in response to pimasertib and cetuximab was paralleled by changes in the phosphorylation of MAPK; thus, whereas the concurrent treatment with cetuximab and pimasertib failed to further decrease the strong inactivation of ERK1/2 imposed by cetuximab in *NRAS*^+/+^ parental cells, the addition of cetuximab fully deactivated the remaining MAPK activity in pimasertib-treated *NRAS*^*Q61K*/+^ cells (Figure 7).

### Real-time monitoring of cell proliferation confirms the synergistic interaction between cetuximab and MEK1/2 inhibition in *NRAS* mutant mCRC cells

**Figure 8:**
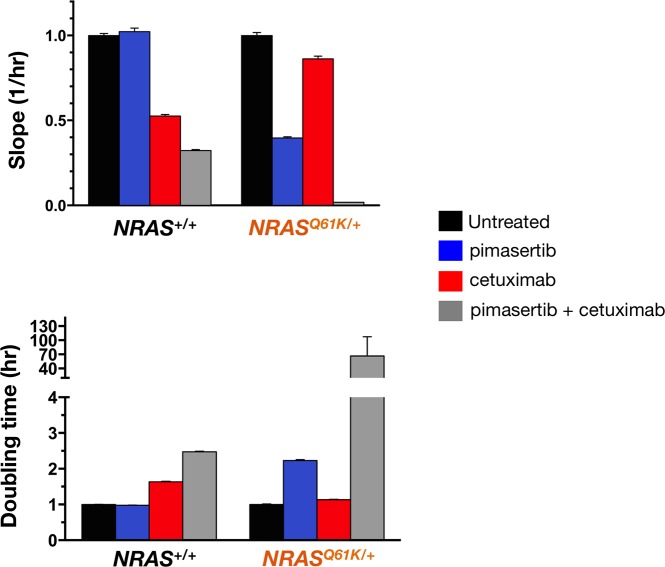
MEK1/2 inhibitor and cetuximab synergistically decrease the proliferation rate of *NRAS* mutant mCRC cells The rate of proliferation was monitored in real-time using the xCELLligence system. Figure shows the rates of proliferation (*top panel*) and cell doubling times (*bottom panel*) in the presence of pimasertib (10 nmol/L), cetuximab (100 μg/mL), or pimasertib + cetuximab as determined by analyzing the growth curves shapes of *NRAS*^+/+^ (*left panels*) and *NRAS*^*Q61K*/+^ between the 24 and 96 h hour interval. Results are shown as mean (*columns*) ± SD (*error bars*) from at least two experiments in which triplicate wells were analyzed.

A limitation in the use of MTT reduction and cell death ELISA analysis is that because they are end-point assays, they offer only a snapshot of what is occurring. We therefore used an impedance-based RTCA platform to capture real-time kinetic data on cell growth after treatment with cetuximab and/or pimasertib. This technology generates a label-free environment for the cancer cells and can accurately inform about the characteristics of a cancer cell's response to a treatment, without the use of toxic/end-point assays leading to the termination of the experiment. Cell proliferation rates (Figure 8, *top panel*) and cell doubling times (Figure 8, *bottom panel*) for *NRAS*^+/+^ and *NRAS*^*Q61K*/+^ cells cultured with or without pimasertib, cetuximab, or the combination pimasertib + cetuximab, were calculated as the slope of the growth curve of best fit from cell index recordings within a given time frame (i. e., between the 24 and 96 h interval). Cell proliferation in the presence of cetuximab was significantly higher in *NRAS*^*Q61K*/+^ cells than in *NRAS*^+/+^ cells, thus confirming the refractoriness of *NRAS*^*Q61K*/+^ cells to the anti-proliferative effects of cetuximab. Co-treatment with pimasertib and cetuximab markedly reduced the cell proliferation rate of *NRAS*^*Q61K*/+^ cells, revealing a synergistic effect between MEK1/2 inhibition and cetuximab in *NRAS*^*Q61K*/+^ cells but not in *NRAS*^+/+^ cells (Figure 8, *top panel*). Accordingly, a drastic > 70-fold increase in cell doubling time was observed in *NRAS*^*Q61K*/+^ cell populations co-treated with the MEK1/2 inhibitor pimasertib and cetuximab (Figure 8, *bottom panel*).

## DISCUSSION

Anti-EGFR monoclonal antibodies including cetuximab and panitumumab have improved the prognosis of a subgroup of patients with mCRC highly dependent on EGFR signaling. Retrospective studies based on prospectively randomized clinical trial data (prospective-retrospective analysis) have confirmed that the best tool that oncologists have for selecting mCRC patients for anti-EGFR antibody treatment is to exclude those with *KRAS* or *NRAS* mutations. Accordingly, international clinical guidelines recommend restricting the use of these drugs to mCRC patients with *KRAS* and *NRAS* wild-type tumors [24–26]. However, whereas *RAS* mutations represent the most important predictive biomarker of resistance to anti-EGFR therapy in mCRC, and the only one approved for clinical use, there is an unmet need for new therapeutic strategies for *RAS* mutant tumors. Unfortunately, no therapy is available to specifically inhibit mutant *RAS* and current approaches mostly focus on inhibiting proteins of downstream signaling pathways. Our study leads us to propose that MEK1/2 inhibitors, in combination with cetuximab, might have clinical utility in *NRAS*-mutant mCRC patients.

There are no known human colorectal cancer cell lines that express mutationally activated *NRAS*. To circumvent this problem, we exploited a recently developed cell culture system that accurately recapitulates the genetic changes present in human mCRC. We used an isogenic pair of mCRC cell lines in which homologous recombination has been used to knock-in *Q61K*, the most common driver mutation in *NRAS*. This experimental approach enables the identification of different outputs from wild-type and mutant *NRAS* without the confounding effects of significant differences in genetic background or the possibility of creating “artificial phenotypes” by forced overexpression of oncogenic forms. Thus, our study aimed to provide an unbiased global screen of signaling pathways downstream of endogenous oncogenic *NRAS* in response to the anti-EGFR monoclonal antibody cetuximab.

Knock-in of the *Q61K* mutant allele rendered SW48 cells, one of the most highly sensitive CRC cell lines to cetuximab treatment [21], fully refractory to the growth inhibitory activity of this antibody. Because SW48 cells should be viewed as a “quadruple wild-type” model for *KRAS*, *BRAF*, *NRAS*, and *PIK3CA* genes, these findings formally prove that a mono-allelic activating mutation of *NRAS* is sufficient to causally drive cetuximab resistance in EGFR-dependent mCRC cells.

Despite the fact that mutant forms of *RAS* are thought to engage multiple downstream effectors to transmit their oncogenic signals, cetuximab-unresponsive ERK1/2 phosphorylation was the sole biomarker distinguishing cetuximab-refractory *NRAS*^*Q61K*/+^ from cetuximab-sensitive *NRAS*^+/+^ cells in our assays. These results strongly suggest that *NRAS*-mutant mCRC cells exhibit intrinsic refractoriness to cetuximab by being able to efficiently channel signaling through the MEK/ERK pathway. Our findings are in accord with an earlier landmark study showing that *NRAS* mainly signals through ERK to activate an anti-apoptotic phenotype, and suggesting that MEK1/2 inhibitors could be highly efficacious for patients with *NRAS*-mutant primary colorectal cancer [13]. But, it remained unclear in that study whether ERK1/2 activation was the particular effector pathway in *NRAS* mutant cells instrumental to mediate the cetuximab-resistant phenotype in mCRC. To explore this possibility, we used a panel of highly specific MEK inhibitors that have entered the laboratory and the clinic [17–20]. We noted, however, that pharmacological suppression of MEK1/2 activity failed to block the growth of cetuximab-resistant *NRAS* mutant cells. Although the finding that *NRAS*^*Q61K*/+^ cells were significantly more resistant to pharmacological suppression of MEK1/2 was unexpected [12], we reasoned that ERK1/2 dependency associated with drugs inhibiting EGFR pathways, such as cetuximab, might become apparent only when the receptor itself was simultaneously targeted. Accordingly, concomitant blockade of EGFR with cetuximab and of MEK1/2 with pimasertib, a selective allosteric MEK inhibitor, was a highly effective combination to reduce the survival of *NRAS*^*Q61K*/+^ cells. The synergistic pro-apoptotic effect of MEK1/2 and EGFR inhibition imply that concurrent targeting of MEK and EGFR signaling may constitute a rational strategy for the treatment of *NRAS* mutant mCRC.

While the lack of phosphorylated MAPK protein inhibition as a finding in primary resistance to cetuximab in *NRAS*-mutant mCRC cells concurs with earlier findings showing that primary and acquired resistance to EGFR inhibitors converge on the MAPK pathway [27, 28], the discovery that mCRC cells with resistance to cetuximab through an activating *NRAS* mutation display constitutive activation of MEK, but are only modestly affected by MEK inhibitors, was intriguing. By surveying intracellular signaling pathways with pimasertib and cetuximab alone or in combination, we found that the intensity of adaptive responses engaging feedback loops (e.g., activation of the AKT pathway) might determine the success of tumoricidal responses in *NRAS*^*Q61K*/+^ cells challenged with cetuximab. Although further investigations are needed to elucidate the precise biochemical mechanisms underlying the remarkably stronger effectiveness of the MEK-EGFR inhibitory combination in *NRAS*-mutant, but not in *NRAS*-WT, mCRC cells [22, 23], it seems that AKT or ERK is activated as a counterpart signal when cells are exposed to MEK1/2 or EGFR inhibitors, respectively. Thus, whereas pimasertib inhibits the MEK/ERK pathway but induces an evident anti-apoptotic activation of AKT in *NRAS*^+/+^ cells, cetuximab does not inhibit the MEK/ERK pathway, but is able to abrogate the weaker activation of AKT in pimasertib-treated *NRAS*^*Q61K*/+^ cells. Because the ability of AKT to promote survival is dependent on and proportional to its kinase activity, the inverse correlation between AKT activation and PARP cleavage might explain the exacerbated cell death response to pimasertib and cetuximab in *NRAS*^*Q61K*/+^ but not in wild-type mCRC cells. Indeed, because real-time monitoring of cell proliferation confirmed that cetuximab co-treatment drastically augmented the growth inhibitory effects of pimasertib in *NRAS* mutant but not wild-type cells, it is reasonable to suggest that cetuximab might be a synthetic lethal agent [29, 30] in combination with MEK1/2 inhibitors for the *NRAS* mutant subgroup of mCRC.

Overall, our results demonstrate that *NRAS*-mutant mCRC cells display sustained activation of MEK/ERK that persists after cetuximab-induced EGFR blockade. Our report confirms the notion that, regardless of the gene/mutation that confers resistance to cetuximab (*KRAS*, *BRAF*, *NRAS*, *MET*, *HER2*), the net signaling output appears to invariably involve the constitutive phosphorylation of MEK/ERK. Although these data provide a rationale for overcoming primary *NRAS* mutation-driven primary (*de novo*) and secondary (acquired) resistance to cetuximab using MEK inhibitors, we found that pharmacological inhibition of MEK1/2 alone failed to more efficiently impair the growth of cetuximab-refractory *NRAS*-mutant cells. Circumvention of cetuximab refractoriness in *NRAS* mutant mCRC cells seems to be dependent on the concomitant blockade of MEK and EGFR, and this was required to fully curb the proliferation and survival of cetuximab-refractory *NRAS*-mutant cells. Whereas individual agents produced little or no induction of apoptosis, concomitant blockade of MEK1/2 and EGFR severely impaired the growth of *NRAS*-mutant cells by promoting high levels of apoptotic cell death, thus corroborating that a double hit (MEK-EGFR) is required to achieve a more effective inhibition of key intracellular signals and compensatory feedbacks for cell survival and proliferation in *NRAS*-mutant mCRC cell populations.

mCRC patients with a mutant *NRAS* gene are in dire need of better treatment options. Our study provides pre-clinical evidence for promising synergies between the EGFR antagonist cetuximab and drugs targeting MEK1/2 in *NRAS* mutant mCRC. The synthetic lethality approach may be particularly valuable for the treatment of mCRC with “undruggable” oncogenic drivers such as *NRAS*, a currently considered cetuximab-ineligible population of mCRC patients.

## MATERIALS AND METHODS

### Cell lines

The X-MAN™ isogenic cell lines SW48 *NRAS*-*WT PI3K*α*-WT* (*NRAS*^+/+^ *PI3K*α^+/+^), SW48 *NRAS*^*Q61K*/+^ (Cat# HD 103-017), and SW48 *PI3K*α^*H1047R*/+^ (Cat# HD 103-005), were purchased from Horizon Discovery Ltd. (Cambridge, UK) and maintained according to the supplier's recommendations in RPMI 1640 with 2 mmol/L L-glutamine, 25 mmol/L sodium bicarbonate and 10% FBS.

### Drugs and materials

Cetuximab was kindly provided by the Hospital Universitari de Girona Dr. Josep Trueta Pharmacy (Girona, Spain). Cetuximab was solubilized using 10 mmol /L NaCl in phosphate buffered saline (PBS) at pH 7.2 in bacteriostatic water for injection purposes (stock solution was 2 mg/mL), stored at 4°C and used within 1 month of preparation. MEK1/2 inhibitors binimetinib, trametinib, selumetinib, and pimasertib were purchased from Selleck chemicals Inc. (Houston, TX). MEK1/2 inhibitors were dissolved in sterile dimethylsulfoxide (DMSO) and a 10 mmol/l stock solution was prepared and stored in aliquots at −20°C. Working concentrations were diluted in culture medium prior each experiment. ERK1/2 and phospho-ERK1/2 rabbit polyclonal antibodies were from Cell Signaling Technology (Beverly, MA).

### Metabolic status assessment (MTT-based cell viability assays)

Cell viability was determined using a standard colorimetric MTT (3-4,5-dimethylthiazol-2-yl-2,5-diphenyl-tetrazolium bromide) reduction assay. For each treatment, cell viability was evaluated as a percentage using the following equation: (OD_570_ of the treated sample/OD_570_ of the untreated sample)×100.

### Phospho-proteome profiling

Cells were rinsed with cold-PBS and immediately solubilized in NP-40 lysis buffer (1% NP-40, 20 mmol/L Tris-HCl (pH 8.0), 137 mmol /L NaCl, 10% glycerol, 2 mmol/L EDTA, 1 mmol/L sodium orthovanadate, 10 *μ*g/mL aprotinin and 10 *μ*g/mL leupeptin) by rocking the lysates gently at 4°C for 30 min. Following centrifugation at 14 000 × *g* for 5 min, supernatants were transferred to a clean test tube and protein concentrations were determined using the BCA Protein kit (Pierce, Rockford, IL). Lysates were diluted and incubated with Human Phospho-MAPK and Human Phospho-Kinase (Proteome Profiler; R&D Systems; Minneapolis, MN) as per the manufacturer's instructions. In this method, capture and control antibodies were spotted in duplicate on nitrocellulose membranes. Briefly, the membranes were blocked with 5% bovine serum album (BSA) in TBS (0.01 mol/L Tris-HCl, pH 7.6) for 1 h. Membranes were then incubated with 750 *μ*g of total protein. After extensive washing with TBS including 0.1% v/v Tween-20, three times for 5 min, to remove unbound materials, membranes were incubated with HRP-conjugated secondary antibodies for 2 h at room temperature (RT). Unbound HRP antibody was washed out with TBS/ 0.1% v/v Tween-20. Finally, array data were developed on X-ray film using a chemiluminescence detection system (Amersham Life Sciences, Piscataway, NJ). Densitometry analyses of the scanned phospho-arrays were carried out using Carestream Molecular Imaging Software.

### Apoptosis assay

Apoptosis was assessed using the Cell Death Detection ELISA^PLUS^ Kit from Roche Diagnostics (Barcelona, Spain). Briefly, cells (5-10 × 10^3^ cells per well) were grown in 96-well plates and treated in triplicate with the indicated doses of MEK1/2 inhibitors, cetuximab, or MEK1/2 inhibitors + cetuximab for 72 h. Pelleted cells were treated with lysis buffer for 30 min at RT. Anti-histone biotin and anti-DNA peroxidase antibodies were then added to each well followed by incubation at RT for 2 h. After three washes, the peroxidase substrate was added to each well, and the plates were read at 405 nm at multiple time intervals. The enrichment of histone-DNA fragments in treated cells was expressed as the fold increase in absorbance relative to control (vehicle-treated) cells using the following formula: [*A*_405_−*A*_490_]_TREATED_/ [*A*_405_−*A*_490_]_UNTREATED_.

### PathScan sandwich immunoassay

The PathScan^®^ Intracellular Signaling array kit (Cell Signaling Technology, #7323) was used. Briefly, overnight serum-starved cells cultured in the presence of graded concentrations of pimasertib with or without 100 μg/mL cetuximab were washed with ice-cold 1× PBS and lysed in 1× Cell Lysis buffer. The Array Blocking Buffer was added to each well followed by incubation for 15 min at RT. Subsequently, the lysate was added to each well and incubated for 2 h at RT. After washing, the detection antibody cocktail was added to each well and incubated for 1 h at RT. Horseradish peroxidase (HRP)-linked streptavidin was added to each well and incubated for 30 min at RT. The slide was then covered with ECL Clarity (Bio-Rad) and images were captured.

### Immunoblotting

Cells were washed two times with PBS and then lysed in buffer [20 mmol/L Tris (pH 7.5), 150 mmol/L NaCl, 1 mmol/L EDTA, 1 mmol/L EGTA, 1% Triton X-100, 2.5 mmol/L sodium pyrophosphate, 1 mmol/L β-glycerolphosphate, 1 mmol/L Na_3_VO_4_, 1 μg/mL leupeptin, 1 mmol/L phenylmethylsulfonylfluoride] for 30 min on ice. The lysates were cleared by centrifugation (15 min at 14.000 rpm, 4°C). Protein content was determined against a standardized control using the Pierce protein assay kit (Rockford, IL). Equal amounts of protein (50 μg) were heated in SDS sample buffer (Laemmli) for 10 min at 70°C, subjected to electrophoresis on 10% SDS-PAGE, and transferred to nitrocellulose membranes. Nonspecific binding on the nitrocellulose filter was minimized by blocking for 1 h at room temperature (RT) with TBS-T [25 mmol/L Tris-HCl, 150 mmol/L NaCl (pH 7.5), and 0.05% Tween 20] containing 5% (*w/v*) nonfat dry milk. The treated filters were washed in TBS-T and then incubated overnight at 4°C with primary antibody in TBS-T/5% bovine serum albumin (BSA). The membranes were washed in TBS-T, horseradish peroxidase-conjugated secondary antibodies (Jackson Immuno Research, West Grove, PA) in TBS- containing 5% (*w/v*) nonfat dry milk were added for 1 h, and immunoreactive bands were visualized with the Clarity™ Western ECL Substrate (Bio-Rad).

### Real-time cell growth rates

Proliferation was measured by using the xCELLigence RTCA DP Instrument (ACEA Biosciences, San Diego). Cells were plated at 20,000 cells/well in fresh medium in 200 μL in an E-plate 16. Initial attachment and growth were continuously monitored for approximately 24 h at 37°C and 5% CO_2_ for stabilization. Then, 100 μL of medium was removed from each well and replaced with fresh medium with or with compounds to achieve the appropriate final concentrations. The plate remained in the RTCA Station for 96 h and cell proliferation was monitored in real time and plotted using RTCA software. Three biological replicates were evaluated in each experiment. Cellular growth rate was determined by the slope of the growth curve using the RTCA Software Package 1.2, which permits normalization to any time point, and results can be directly viewed in the software window. We conducted the normalization at one time point before the treatment.

### Statistical analysis

All observations were confirmed by at least three independent experiments. Data are presented as mean ± SD. Two-group comparisons were performed using Student's *t* test for paired and unpaired values. Comparisons of means of ≥ 3 groups were performed by ANOVA, and the existence of individual differences, in case of significant *F* values at ANOVA, tested by Scheffé's multiple contrasts. *P* values < 0.01 were considered to be statistically significant (denoted as^*^). All statistical tests were two-sided.

## SUPPLEMENTARY MATERIAL


