# Supplementary material for: Synthetic lethal interaction of cetuximab with MEK1/2 inhibition in NRAS-mutant metastatic colorectal cancer
Source: Oncotarget. 2016 Sep 12;7(50):82185–99. doi: 10.18632/oncotarget.11985 (PMC5347684; doi:10.18632/oncotarget.11985)
Supplement: Supplementary file 1 [file oncotarget-07-82185-s001.pdf]

Synthetic lethal interaction of cetuximab with MEK1/2 inhibition in *NRAS*-mutant metastatic colorectal cancer

Supplementary Material

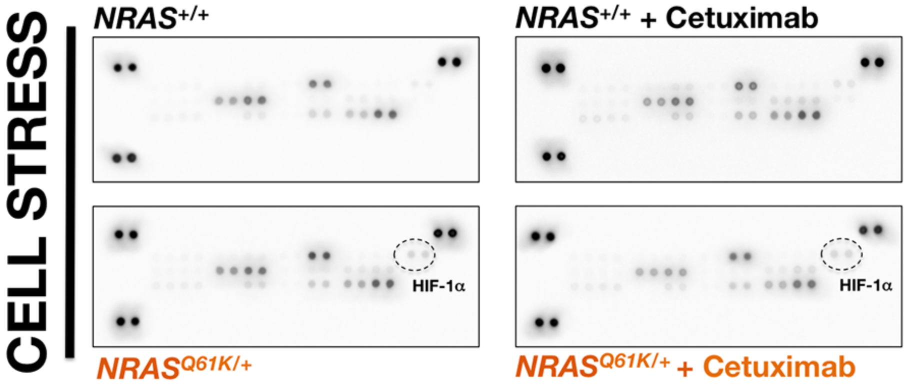

Supplemental Figure 1. *NRAS* mutant mCRC cells exhibit a slight, constitutive activation of HIF-1α in response to cetuximab. Total cell lysates (750 μg) from *NRAS*$^{+/+}$ and *NRAS*$^{Q61K/+}$ cells before and after treatment with 100 μg/mL cetuximab (48 h) were incubated with membranes of the phosphoproteomic platform human Cell Stress Human Proteome Profiler Array (26 cell stress-related proteins), as per the manufacturer's instructions (Proteome Profiler; R&D Systems). Figure shows representative phosphoproteome analyses that were developed on X-ray film following exposure to chemiluminiscent reagents. Equivalent results were obtained in two independent experiments.
